# Genome-Wide Association Study Identifies Genetic Variants Associated with Rotator Cuff Tear—A Pilot Study

**DOI:** 10.3390/diagnostics12102497

**Published:** 2022-10-15

**Authors:** Hyun-Ju An, Jae-Hwa Kim, Siyeong Yoon, Junwon Choi, Jeongmo Koo, Soonchul Lee

**Affiliations:** 1 Department of Orthopaedic Surgery, CHA Bundang Medical Center, CHA University School of Medicine, 335 Pangyo-ro, Bundang-gu, Seongnam-si 13488, Gyeonggi-do, Korea; 2 Department of Molecular Science and Technology, Ajou University, 206 World cup-ro, Yeongtong-gu, Suwon-si 16499, Gyeonggi-do, Korea

**Keywords:** rotator cuff tear, single nucleotide polymorphism, genome-wide association study

## Abstract

A rotator cuff is a muscle and tendon surrounding the shoulder joint, and a rotator cuff tear can be caused by overuse or injury, which leads to great pain in affected individuals. However, rotator cuff tear is a multifactorial process whose underlying mechanism is still unclear. Many previous studies have suggested an important role of genetic predisposition, such as single-nucleotide polymorphisms (SNPs), in explaining the genesis of tendinopathy. This study aimed to identify specific genes or genetic variants associated with rotator cuff tears by performing a genome-wide association study (GWAS) using an independent case of rotator cuff tears. GWAS was performed using data from CHA Bundang Medical Center with 20 cases of rotator cuff tears, and 20 cases of healthy controls genotyped on the Illumina HiSeq 2500. Tests of association were performed using the Burrows–Wheeler Aligner (BWA) software at 284,246 SNPs. Data were filtered based on sequence ontology, minor allele frequency, and Hardy–Weinberg equilibrium values, and SNPs were considered significant if the *p*-value was <0.05. The tests of association revealed more than 20 significantly associated SNPs. SNPs showing the highest significance occurred in candidate genes, including *LAIR2* (rs2287828, OR 9.116, *p*-value 5.49 × 10^−4^) on chromosome 19 and *CRIPAK* (rs9328733, OR 6, *p*-value 1.11 × 10^−3^) and *REST* (rs2228991, OR 8.222, *p*-value 1.20 × 10^−3^) on chromosome 4. This study attempted to identify genetic variants influencing rotator cuff tears through a genome-wide association study using a dense set of SNPs. More than 20 SNPs were significantly associated with rotator cuff tears. The major limitation of this study is that it was conducted on a small study group and requires further validation. Nevertheless, the identification of potential genetic variants related to rotator cuff injury would aid in the early detection of individuals at risk for the development of tendinopathy and will provide insight into future gene therapies.

## 1. Introduction

Rotator cuff (RC) tears are among the most common musculoskeletal diseases [1]. They affect up to 50% of the elderly population over the age of 50 years and often lead to shoulder pain and loss of function [2,3]. Though aging, smoking, hypercholesterolemia, genetic predisposition, and shoulder overuse are known risk factors for RC tears [3,4], their pathophysiology is not entirely understood. Extrinsic factors, such as mechanical trauma, and intrinsic factors, such as increased tendon cell apoptosis, excessive fat infiltration, and decreased nutrient vessels, explain some of the underlying mechanisms [5,6,7]. Moreover, genetic variants associated with various intrinsic and extrinsic factors have been proposed to explain the multifactorial processes underlying RC tears [8]. Notably, single nucleotide polymorphisms (SNPs) have been used to understand the genesis of rotator cuff lesions [9].

The investigation of six candidate genes (*DEFB1*, *DENND2C*, *ESRRβ*, *FGF3*, *FGF10*, and *FGFR1*) involved in the repair and degenerative processes of rotator cuff disease by Motta et al. indicated the presence of 23 SNPs [2]. Genetic polymorphisms of *MMP-1* and *MMP-3* [10] and 15 SNPs in *TNC* are also implicated in RC tears [11]. In addition, having a risk allele at rs71404070(A/T), an SNP located next to cadherin8, was found to increase the chance of rotator cuff injury in a genome-wide association screening [12]. However, previous studies on SNPs associated with RC tears are lacking, and understanding the pathogenesis of RC tears remains challenging. Identifying possible genetic variants associated with RC tears may further outline the underlying biological causes, thereby improving the potential to diagnose, treat, and prevent shoulder diseases.

Recently, genome-wide association studies (GWAS) have emerged as a promising tool for identifying the relationship between disease and genetic susceptibility [13]. This tool evaluates millions of SNPs from the genome by performing case-control association analysis and screens SNPs in the whole genome to identify susceptible sites and regions associated with diseases [14].

This study aimed to identify SNPs associated with RC tears using GWAS to compare healthy subjects with patients with RC tears. Through this study, we newly identified SNPs of genes LAIR2, CRIPAK, and REST that were significantly altered in patients with rotator cuff tears. A better understanding of the loci related to rotator cuff tears would help to verify genetic markers to predict the risk of tendon injury and prevent the disease.

## 2. Materials and Methods

### 2.1. Ethics Statement

The present study was approved by the Institutional Review Board of CHA Bundang Medical Center, South Korea (No. CHAMC 2019-09-029-002). All participants provided written informed consent for inclusion in the whole-exome sequencing analysis.

### 2.2. Study Population

A total of 20 patients diagnosed with RC tears and 20 healthy blood donors were recruited from 2017 to 2019 at the CHA Bundang Medical Center. WES was performed on genomic DNA extracted from blood samples. Patients in the RC tear group were diagnosed with a full-thickness RC tear and underwent surgical treatment. The condition was evaluated by detailed history-taking with physical examination and magnetic resonance imaging (MRI) (Signa Architect 3.0T; General Electronic Healthcare, Milwaukee, WI, USA). MRI was used to determine the RC tear size. The non-RC tear group had no RC tears and did not undergo previous surgery related to RC tears. The group visited the orthopedic clinic during the study period because of minor trauma (abrasions, shallow lacerations, and contusions) in all areas except the shoulder region. Of the 40 subjects enrolled in the study, 20 were diagnosed with RC tears (RC tear group), while 20 healthy individuals without RC tears comprised the control group. In the RC tear group, there were 15 men and 5 women. The healthy control group included 9 men and 11 women. The mean ages of the control group and the RC tear group were 57.15 ± 13.94 and 62.50 ± 7.48, respectively, which did not show a statistically significant difference. Other parameters, including sex, medical history, and laboratory characteristics, were not significantly different between the groups (Table 1).

### 2.3. Genomic DNA Extraction and Exome Sequencing

WES (Whole Exome Sequencing) was performed using genomic DNA samples extracted from 20 patients with RC tears and 20 healthy controls. Under sterile conditions, 10 mL of venous blood was collected from the jugular vein of the subject milch animals in a 15-mL polypropylene centrifuge tube containing 0.5 mL of 0.5 M EDTA solution, which acts as an anticoagulant. The tube was tightly capped and shaken gently to facilitate the thorough mixing of blood with the anticoagulant. The tubes containing blood samples were transported to the laboratory in an icebox containing ice packs and were kept in the refrigerator, maintaining the temperature at −20 °C till the DNA isolation. Genomic DNA was extracted using the ChargeSwitch gDNA Purification Kits (Thermo Fisher Scientific, Waltham, MA, USA). The quality and quantity of the isolated genomic DNA were evaluated using UV–vis spectrophotometer (Biophotometer Plus, Eppendorf, Hamburg, Germany). DNA sample exons were captured using the SureSelect Human All Exon V5 Kit (Agilent Technologies Inc., Santa Clara, CA, USA). Each captured library was sequenced at Macrogen (Seoul, South Korea) using an Illumina HiSeq 2500 (Illumina Inc., San Diego, CA, USA), according to the manufacturer’s protocol.

### 2.4. Read Mapping and Variant Analysis

Burrows–Wheeler Aligner (BWA) software was used to align the sequence reads to the human genome reference sequence (GRCh37) [15]. The alignment information was stored in the BAM files. Duplicate reads were marked with the command MarkDuplicates (https://broadinstitute.github.io/picard/, accessed on 2 January 2022) from Picard. Local realignment, base quality score recalibration, variant calling, joint genotyping, and variant quality score recalibration were implemented using the Genome Analysis ToolKit (GATK) v3.4 (http://software.broadinstitute.org/gatk/, accessed on 2 January 2022). All samples were annotated using SnpEff v4.1 [16] to classify the variants.

### 2.5. Variant Filtering

After WES, 284,246 SNPs were identified in 20 RC tear and 20 control groups. Variant filtering was performed based on the following criteria. (1) SNPs with cryptic relatedness and sex discrepancy were excluded. (2) We filtered out SNPs with a minor allele frequency (MAF) < 0.05, and a Hardy–Weinberg equilibrium (HWE) *p*-value < 0.05. (3) We selected variants with an association test *p*-value < 0.05. Finally, 5648 SNPs were included in the analysis. Sequence ontology terms (http://asia.ensembl.org/info/genome/variation/prediction/predicted_data.html, accessed on 7 January 2022) were used for filtering.

### 2.6. Statistical Analysis

All statistical analyses were performed using PLINK software v1.07 (https://zzz.bwh.harvard.edu/plink/, accessed on 7 January 2022) [17] and free R 3.6.2 (https://www.r-project.org/, accessed on 7 January 2022) software. To identify the relationship between categorical variables and RC tears, Pearson’s chi-square test was performed for sex and history of osteoporosis, diabetes mellitus, hypertension, and hyperlipidemia. The Student’s *t*-test was used when two continuous dependent variables were compared. SNP allele frequencies were compared between the RC tear group and controls using Fisher’s exact test to identify SNPs associated with RC tears.

## 3. Results

**Figure 1:**
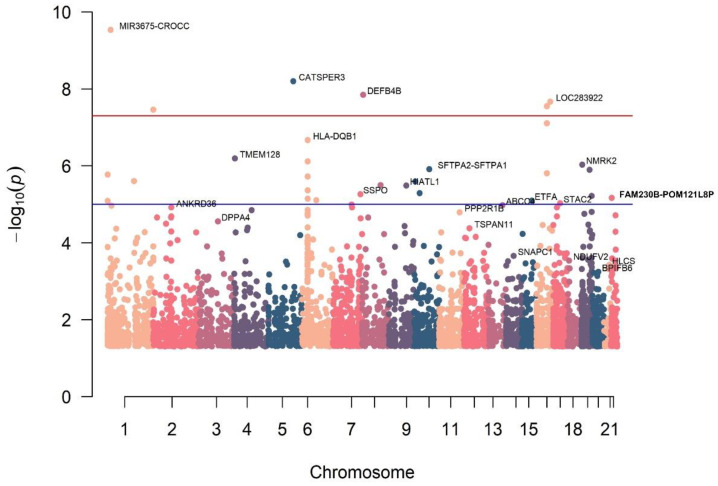
Manhattan plot of the results from the GWAS analysis of RC. The –log_10_ *p* values for association with RC tear for 5638 SNPs from GWAS are plotted by genomic position with chromosome number listed across the bottom. The y-axis shows the –log_10_ *p* values for association with RC tear. SNPs with the lowest *p* values in each chromosome are annotated with the nearest gene name. Red line represents the genome-wide significance threshold* of *p*-value = 5.00 × 10^−^^8^, while the blue line corresponds to the suggestive threshold^†^ of *p*-value = 1.00 × 10^−5^. All data met genome-wide significance criteria with a *p*-value of less than 0.05. * Ref. [16]; ^†^ Ref. [17].

The *p*-values for all SNPs analyzed are shown in the Manhattan plot (Figure 1). Among the 5638 SNPs analyzed, SNP rs141922545 (nearest gene name: *MIR3675*-*CROCC*) was most significantly associated with RC tears (*p* = 2.93 × 10^−^^10^). SNP rs143819145 (*CATSPER3*) and rs71509106 (*DEFB4B*) were second and third, respectively, and were significantly associated with RC tears (*p* = 6.32 × 10^−^^9^, *p* = 2.93 × 10^−^^8^, respectively).

**Figure 2:**
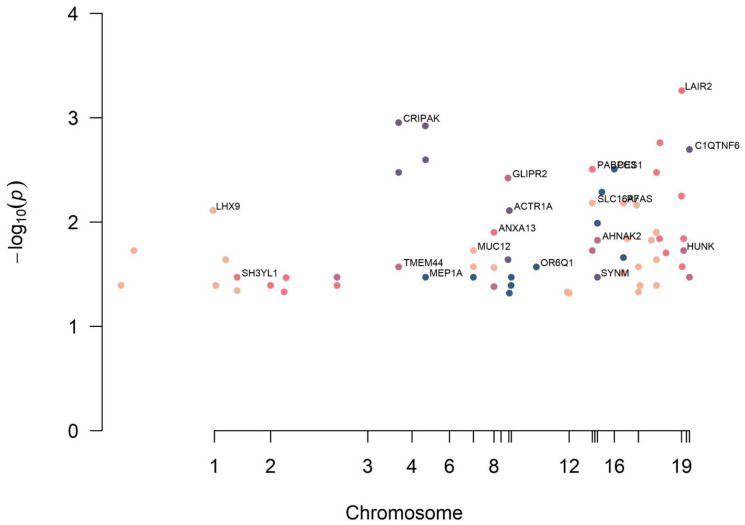
Manhattan plot of the results from the GWAS analysis of RC after filtering. The –log_10_ *p* values for association with RC tear for filtered SNPs data from GWAS are plotted by genomic position with chromosome number listed across the bottom. The y-axis shows the –log_10_ *p* values for association with RC tear. SNPs with the lowest *p* values in each chromosome are annotated with the nearest gene name. All data met genome-wide significance criteria with a *p*-value of less than 0.05.

After filtering the GWAS data with sequence ontology, MAF, and HWE, the 20 SNPs most significantly associated with RC tears were identified in the order of lowest to highest *p* values (Table 2). The Manhattan plot of the filtered data is shown in Figure 2, along with the genetic positions on each chromosome. SNP rs2287828 (*LAIR2*) was most significantly associated with RC tears, with an odds ratio over nine folds (*p* = 5.49 × 10^−^^4^). SNP rs9328733 (*CRIPAK*) and rs2228991 (*REST*) were second and third, respectively, and were significantly associated with RC tears (*p* = 1.11 × 10^−^^3^, *p* = 1.20 × 10^−^^3^, respectively).

## 4. Discussion

Although the human DNA sequence of 3.2 billion bases is more than 99.9% identical, polymorphisms can affect the biological variation of individuals and the genesis of diseases [2,18]. Genetic factors are known to be intrinsic risk factors for the etiology of rotator cuff injuries [19]. Therefore, we hypothesized that individual susceptibility to rotator cuff tears might result from polymorphisms in specific genes related to musculoskeletal degeneration. In this study, more than 20 SNPs were identified in the patients with RC tears. Our results demonstrate that SNPs in various genes with different functions are associated with RC tears.

In this study, we found more than 20 SNPs in RC tear patients. Since the sample size of the study was small, the number of cases = 20, and the number of controls = 20, the sample power was calculated using a genetic power calculator. As a result, we confirmed that high risk allele frequency (A) = 0.25, prevalence = 0.2, genotypic relative risk Aa = 1.27, genotypic relative risk AA = 3.83, alpha = 0.05, to reach 80% power. These results (0.1518) show low power with such a small sample size. So, we performed validation on a large cohort of samples using the whole exome sequencing method. The proportion of quality scores ≥30 points (Q30) was higher than 94.6%, the error rate was 0.001, and the genotype call rate was ≥95%.

The main results can be summarized as follows: (1) *LAIR2* rs2287828 (c.11G > A) is a 5′-UTR variant on chromosome 19, increasing the risk of RC tears more than nine times (OR 9.116); (2) *CRIPAK* rs9328733 (c.284A > G), a missense variant on chromosome 4, elevated the risk of RC tear by six times (OR 6); (3) *REST* rs2228991 (c.1876G > A), a missense variant on chromosome 4, increased the risk of RC tears more than eight times (OR 8.222). *LAIR2* encodes a membrane-bound receptor that binds to collagen and blocks the function of LAIR1, a membranous protein expressed on the immune cell surface that inhibits NK cells, T cells, B cells, monocytes, and dendritic cells [20]. The *LAIR2* SNP variant rs2287828 has been reported to be associated with differential susceptibility to Pemphigus foliaceus [21]. CRIPAK is a negative regulator of Pak1, a serine/threonine kinase involved in cell motility, survival, and mitosis [22]. Genomic alterations of *CRIPAK* are associated with various pathologies, including lung adenocarcinoma, but there are no reports regarding *CRIPAK* rs9328733. SNP rs2228991 in *REST*, a gene in non-neuronal tissues encoding a transcription factor binding to the silencer of neuronal genes, was reported to be associated with an increased risk of colorectal cancer when combined with SNPs in the NFKB1 gene [23]. To the best of our knowledge, none of these 20 SNPs and their nearest genes, including *LAIR2*, *CRIPAK*, and *REST*, have been reported to be associated with rotator cuff tears.

Several studies have investigated gene variations associated with rotator cuff tears. Multiple candidate genes (*DEFB1*, *FGFR1*, *FGFR3*, *ESRRB*, *FGF10*, *MMP-1*, *TNC*, *FCRL3*, *SASH1*, *SA30BP*) and rs71404070 located next to cadherin8 have been implicated in rotator cuff tears [4]. A systematic review by Mousley et al. [24] suggested strong evidence for an association between *MMP-3*, *TNC*, and *ESRRβ* genes and RC tears. Tashjian et al. [25] evaluated 20 previously reported SNPs in 12 genes and confirmed three SNPs (rs1138545, rs72758637, rs7021589; all *p*-values < 0.0024) in the *TNC* gene associated with rotator cuff injury. In addition, *GLCCI1*, another candidate gene potentially leading to chronic inflammatory changes in the rotator cuff, was replicated and validated in the same dataset. However, the involvement of SNPs in specific genes that explain rotator cuff degeneration remains controversial. For example, genetic association studies by Bonato et al. [26], Motta et al. [2], Peach et al. [27], Tashjian et al. [28], Teerlink et al. [29], and Kluger et al. [11] identified 18 SNPs in 10 genes associated with rotator cuff injury, including the *ESRRB* and *TNC* genes. However, an independent study by Roos et al. [12], attempting to validate these previously reported genes with genetic association studies, demonstrated that none of these 18 SNPs showed significant association with rotator cuff injury, despite a powerful sample size of the dataset. Roos et al. explained that the difference in the source of their data compared to previous studies contributed to these irreplicable results. Roos et al. collected data from electronic health records, whereas previous studies used data confirmed by magnetic resonance imaging. Additionally, Kluger et al. [11] and Assunção et al. [10] reported discrepancies in their results. Kluger et al. found no significant difference in genotype and allele frequencies for SNPs in *MMP-1* and *MMP-3* genes, whereas Assunção et al. found a significant association between *MMP-1* and *MMP-3* genes and rotator cuff tears. Assunção et al. explained that the smaller sample size, absence of pairing ages between cases and controls, and no consideration of other risk factors, such as high blood pressure and racial characteristics of the population, caused such discrepancies between the two studies. Further comprehensive studies, including verification through a secondary dataset, are necessary to confirm the association between RC tears and SNPs and their genes to resolve further discrepancies.

One limitation of this study was that the sample size was relatively small for a genome-wide association study. Conducting GWAS using a large and open dataset would be helpful. A second limitation is that this study only evaluated subjects from an Asian group, and the effects on other ethnic backgrounds are unknown. Third, this study did not classify tears as traumatic or chronic. Fourth, we only identified associations without verifying the biological mechanisms by which variants in these genes affect rotator cuff tears. As none of these SNPs have been confirmed in a secondary dataset, an additional study with a robust separated set of cases is needed. Further studies, including in vitro transfection assays and analysis of expression changes at the tissue level, would help confirm the results and verify the underlying biological mechanism for the association between the variation in these genes and rotator cuff tear. Fifth, we did not consider the effects of other risk factors for rotator cuff injury, such as smoking. As rotator cuff tear is a multifactorial process, potential confounding variables and various risk factors should be considered in future research.

## 5. Conclusions

In conclusion, we conducted a GWAS to correlate rotator cuff tears with the presence of SNPs. This study showed an association between the occurrence of rotator cuff tears and several genes, including *LAIR2*, *REST*, and *CRIPAK*. Further research is necessary to validate our findings and identify their potential clinical applications. However, identifying a possible genetic association may further our understanding of the process that leads to rotator cuff tears, aid in the early detection of individuals at risk for the development of tendinopathy, and provide novel clues for future gene therapies.

## Figures and Tables

**Table 1:** Demographic and laboratory characteristics of the study population.

Variable		RC (*n* = 20)	Control (*n* = 20)	OR	*p*-Value
Gender, *n* (%)	Male	15 (75%)	9 (45%)	3.54	0.105 ^†^
	Female	5 (25%)	11 (55%)		
Age		62.50 ± 7.48	57.15 ± 13.94		0.141 *
Past history, *n* (%)					
	Osteoporosis, yes	1 (5%)	2 (10%)	0.48	0.999 ^†^
	Diabetes mellitus, yes	3 (15%)	2 (10%)	1.57	0.999 ^†^
	Hypertension, yes	6 (30%)	1 (5%)	7.76	0.091 ^†^
	Hyperlipidemia, yes	2 (10%)	4 (20%)	0.45	0.661 ^†^
BMD		1.05 (0.2)	-		
WBC (×10^6^/uL)		6.67 ± 2.36	7.62 ± 2.24		0.343 *
RBC (×10^6^/uL)		4.53 ± 0.42	4.54 ± 0.65		0.961 *
Hgb (g/dL)		14.11 ± 1.24	13.46 ± 1.88		0.296 *
Na (mEq/L)		140.74 ± 2.05	141.33 ± 2.18		0.487 *
K (mEq/L)		4.28 ± 0.35	4.31 ± 0.46		0.839 *
CI (mEq/L)		103.16 ± 1.64	104.00 ± 2.12		0.259 *
T.pro (g/dL)		7.00 ± 0.54	10.39 ± 7.81		0.26 *
Albumin (g/dL)		4.38 ± 0.29	4.53 ± 0.28		0.234 *
Ca (mg/dL)		9.46 ± 0.49	9.57 ± 0.55		0.638 *
P (mg/dL)		3.33 ± 0.41	3.27 ± 0.41		0.746 *

* *t*-test, ^†^ Fisher’s exact test; RC: Rotator cuff; OR: Odds ratio; BMD: Bone mineral density; WBC: White blood cell; RBC: Red blood cell; Hgb: Hemoglobin; Na: Sodium; K: Potassium; Cl: Chloride; T.pro: Total protein; Ca: Calcium; P: Phosphorus.

**Table 2:** Identified candidates of genetic variants associated with RC patients.

Chr	Position Type (SNP rs)	A1	A2	Frequency		Candidate Gene	Gene Description	OR	*p*-Value *
				Control (%)	RC (%)				
				*n* = 20	*n* = 20				
19	55,014,124 SNP (rs2287828)	A	G	3 (15)	14 (70)	LAIR2	A membrane-bound receptor that modulates innate immune response.	9.115942029	0.00054903
4	1,388,583 SNP (rs9328733)	A	G	6 (30)	13 (65)	CRIPAK	Negative regulator of PAK1.	6	0.00111562
4	57,796,900 SNP (rs2228991)	A	G	3 (15)	15 (75)	REST	A transcriptional repressor that represses neuronal genes in non-neuronal tissues.	8.222222222	0.00119894
19	9,236,698 SNP (rs111279560)	GATGGT	G	5 (25)	15 (75)	OR7G3	Olfactory receptors protein of a large family of G-protein-coupled receptors (GPCR)	5.333333333	0.00173965
22	37,584,352 SNP (rs13057424)	A	G	6 (30)	15 (75)	C1QTNF6	Complement C1q tumor necrosis factor-related protein 6	5.210526316	0.00202543
4	57,843,295 SNP (rs3733306)	A	C	3 (15)	14 (70)	NOA1	Nitric Oxide-Associated Protein 1	7.4	0.00253432
13	25,671,429 SNP (rs77466429)	T	G	2 (10)	13 (65)	PABPC3	Poly binding protein that cytoplasmic regulatory processes of mRNA metabolism	9.148148148	0.00312074
16	55,862,824 SNP (rs3826192)	T	C	2 (10)	13 (65)	CES1	A member of the carboxylesterase large family.	9.148148148	0.00312074
16	55,862,883 SNP (rs3826190)	A	C	2 (10)	13 (65)	CES1	A member of the carboxylesterase large family.	9.148148148	0.00312074
4	1,389,156 SNP (rs71614972)	T	C	6 (30)	15 (75)	CRIPAK	Negative regulator of PAK1.	5.126984127	0.00334498
19	1,619,350 SNP (rs1052692)	T	C	6 (30)	15 (75)	TCF3	A member of the E protein (class I) family of helix-loop-helix transcription factors.	5.126984127	0.00334498
9	36,135,923 SNP (rs7041851)	C	T	4 (20)	12 (60)	GLIPR2	GLI pathogenesis related 2	6	0.00379856
16	30,021,402 SNP (rs1140239)	T	C	4 (20)	14 (70)	DOC2A	Double C2 domain alpha	5.173913043	0.00517988
19	54,744,387 SNP (rs71263238)	C	T	4 (20)	16 (80)	LILRB3	A member of the leukocyte immunoglobulin-like receptor (LIR) family	4.960784314	0.00564698
12	60,173,356 SNP (rs3763980)	T	C	5 (25)	16 (80)	SLC16A7	A member of the monocarboxylate transporter family.	4.636363636	0.00660224
17	8,161,149 SNP (rs4791641)	T	A	6 (30)	15 (75)	PFAS	Catalyzes biosynthesis of DNA replication, transcription, and energy metabolism	4.636363636	0.00660224
17	35,300,494 SNP (rs35033250)	G	GA	5 (25)	15 (75)	LHX1	A transcription factor of a large protein family which contains the LIM domain,	4.363636364	0.00690291
1	197,901,206 SNP (rs34018572)	CA	C	4 (20)	12 (60)	LHX9	A transcription factor of a large protein family which contains the LIM domain,	5.1	0.00778198
10	104,240,498 SNP (rs12162)	G	A	4 (20)	13 (65)	ACTR1A	A macromolecular complex consisting of subunit of dynactin	5.1	0.00778198
16	20,477,004 SNP (rs59261767)	T	C	3 (15)	12 (60)	ACSM2A	A macromolecular complex consisting of subunit of dynactin	5.938271605	0.01028714

* Fisher’s exact test using allelic model; Chr: Chromosome, A1: Minor allele, A2: Major allele; SNP: Single nucleotide polymorphism, RC: Rotator cuff.

## Data Availability

The authors confirm that the data supporting the findings of this study are available within the article.
